# Serial DNA relay in DNA logic gates by electrical fusion and mechanical splitting of droplets

**DOI:** 10.1371/journal.pone.0180876

**Published:** 2017-07-10

**Authors:** Hiroki Yasuga, Kosuke Inoue, Ryuji Kawano, Masahiro Takinoue, Toshihisa Osaki, Koki Kamiya, Norihisa Miki, Shoji Takeuchi

**Affiliations:** 1 Artificial Cell Membrane Systems Group, Kanagawa Institute of Industrial Science and Technology, Kawasaki, Japan; 2 School of Integrated Design Engineering, Keio University, Yokohama, Japan; 3 Department of Biotechnology and Life Science, Tokyo University of Agriculture and Technology, Tokyo, Japan; 4 Interdisciplinary Graduate School of Science and Engineering, Tokyo Institute of Technology, Yokohama, Japan; 5 Institute of Industrial Science, The University of Tokyo, Tokyo, Japan; 6 Department of Mechanical Engineering, Keio University, Yokohama, Japan; University of the West of England, UNITED KINGDOM

## Abstract

DNA logic circuits utilizing DNA hybridization and/or enzymatic reactions have drawn increasing attention for their potential applications in the diagnosis and treatment of cellular diseases. The compartmentalization of such a system into a microdroplet considerably helps to precisely regulate local interactions and reactions between molecules. In this study, we introduced a relay approach for enabling the transfer of DNA from one droplet to another to implement multi-step sequential logic operations. We proposed electrical fusion and mechanical splitting of droplets to facilitate the DNA flow at the inputs, logic operation, output, and serial connection between two logic gates. We developed Negative-OR operations integrated by a serial connection of the OR gate and NOT gate incorporated in a series of droplets. The four types of input defined by the presence/absence of DNA in the input droplet pair were correctly reflected in the readout at the Negative-OR gate. The proposed approach potentially allows for serial and parallel logic operations that could be used for complex diagnostic applications.

## Introduction

A DNA logic circuit is a form of biological information processing system. Inputs and outputs are often DNA molecules with specific nucleotide sequences. The logic operations are implemented by a set of designed biochemical processes such as DNA hybridization or enzymatic reactions[1–10]. Practically, the logic operation is initiated by mixing the input DNA molecules with the reaction solution in a single tube, and the resulting output phenomena are read out by performing optical[11,12] or electrical measurements[13,14]. Based on previous studies, DNA logic circuits proved their potential for practical applications such as cancer diagnosis based on microRNA analysis and disease-specific DNA sequencing[3,15,16]. In electronics, general logic circuits implement a complex operation simply by connecting minimum units of logic gates (e.g., AND, OR, NOT, NAND, and NOR). However, in DNA logic circuits, performing such complex operations using minimum units is difficult because unintended crosstalk reactions of DNA and enzymes are likely to occur once the molecules are mixed in solution[17,18].

**Fig 1:**
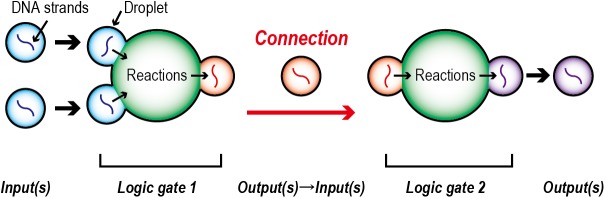
DNA logic gate compartmentalization into a droplet and connection of the gates.

In this study, we develop a multi-step sequential reaction system based on DNA logic circuits that can suppress unintended crosstalk reactions. We compartmentalize each component (i.e., input, output, and logic gate) into a water-in-oil droplet, and connect the units step by step by mixing the droplets (Fig 1). After being incorporated into input droplets, the DNA, whose presence/absence determines the true/false, is relayed to a droplet in which the logic operation occurs. The resulting DNA is subsequently relayed to an output droplet that connects to a second input logic gate. Using this DNA relay mechanism, DNA logic gates could be connected to perform complicated logic operations. Here, we implement a relay mechanism by fusing droplets using pulsed voltage and by mechanically splitting the fused droplet. With this relay mechanism, we develop a Negative-OR (NOR) gate by connecting OR and NOT gates, and examine the reliability of the operation results.

## Working principle

### DNA relay in logic gates via fusion and splitting

**Fig 2:**
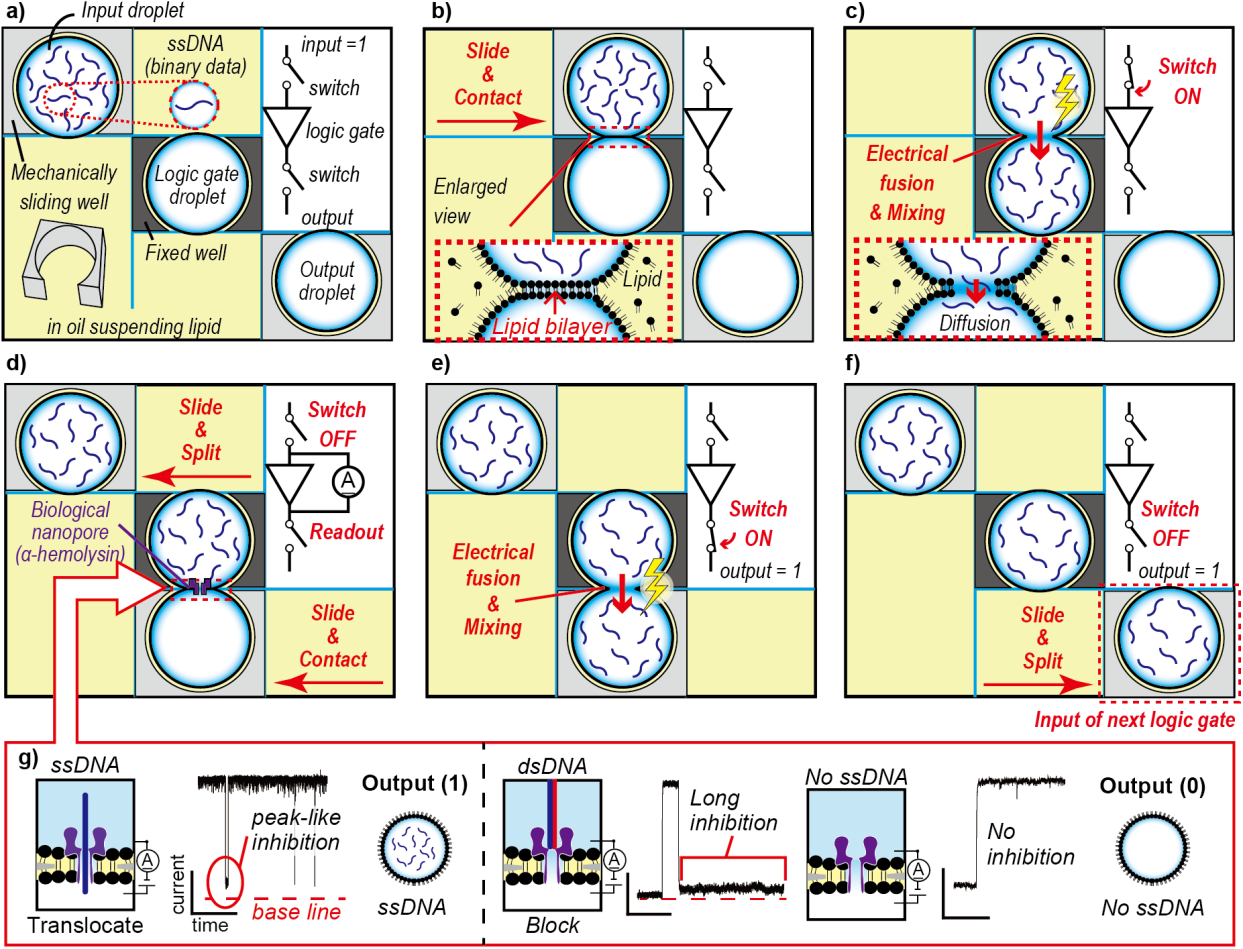
Conceptual diagrams of the DNA relay mechanism. (a–f) Logic operation using the mechanical splitting and electrical fusion of droplets (YES gate). (a) Three aqueous droplets (prepared as input, logic gate, and output) in mechanically sliding and fixed wells. (b) Contact of the input droplet to the logic gate droplet with sliding motion to form a lipid bilayer. (c) Electrical fusion by lipid bilayer rupture and mixing of single-stranded DNA (ssDNA; binary data) over the droplets: switch-on of the input. (d) Contact of the output droplet to the logic gate droplet with sliding motion to form a lipid bilayer and perform nanopore detection. (e) Electrical fusion and mixing of ssDNA (binary data) over the droplets: switch-on of the output. (f) Output droplet split-off from the logic gate droplet for use as the input droplet of the next logic gate. (g) Translocation of ssDNA, blocking by dsDNA, and no inhibition. Observation of peak-like current inhibition implies the translocation of ssDNA and the storage of ssDNA in the output droplet, leading to output 1. Observation of a long current inhibition implies the blocking of α-hemolysin nanopore by dsDNA, and no DNA strand is stored in the logic gate droplet, resulting in output 0. Alternatively, no inhibition implies no ssDNA and, therefore, the output is 0.

In this study, the logic gates were operated via a DNA relay mechanism based on electrical fusion and mechanical splitting. The logic gates were implemented in a Split-and-Contact Method device equipped with electrodes[19]. As shown in Fig 2A, the input, logic gate, and output aqueous droplets are prepared in mechanically sliding and fixed wells. The entire system is immersed in lipid-dispersed oil, thereby covering the droplets with a monolayer of lipid molecules. The binary number 1 (true) or 0 (false) in the operation is defined as the presence or absence of single-stranded DNA (ssDNA) in the droplet, respectively.

Initially, the input droplet, with or without ssDNA, makes contact with the logic gate droplet. The contact between two lipid monolayers on a pair of droplets forms a lipid bilayer membrane (Fig 2B) [20,21]. Then, the lipid bilayer is ruptured by applying pulsed voltage, resulting in the fusion of the droplets and mixing of DNA over the droplets (Fig 2C, switch-on of the logic gate input). The first relay of the binary DNA data is terminated by splitting the input droplet from the logic gate droplet (Fig 2D). As illustrated in Fig 2D, 2E and 2F, the same cycle is performed to relay the binary DNA data from the logic gate droplet to the output droplet (Fig 2F).

### Output readout

The output readout is performed at the interface of the logic gate and output droplets. In these droplets, electrodes are installed to measure the current. As shown in Fig 2D, α-hemolysin (αHL) is reconstituted to form a nanopore in the lipid bilayer between these droplets. The nanopores and the lipid bilayer provide selectivity between the droplets (Fig 2G). ssDNA, which is negatively charged, is transported through the nanopore with the help of a potential gradient. αHL nanopore detection determines the type of output readout because the pore size allows for the translocation of ssDNA but blocks double-stranded DNA (dsDNA) (Fig 2G)[22,23]. The key advantage of the nanopore readout is the label-free electrical detection of DNA at the single-molecule level[23]. The resultant DNA (i.e., ssDNA, dsDNA, or no DNA, depending on the input) is distinguished by the αHL nanopore. It should be noted that the nanopore-based detecting system is able to identify DNA/RNA strand with a specific base sequence in a solution where other strands are mixed [15,24,25].

#### NOR operation

**Fig 3:**
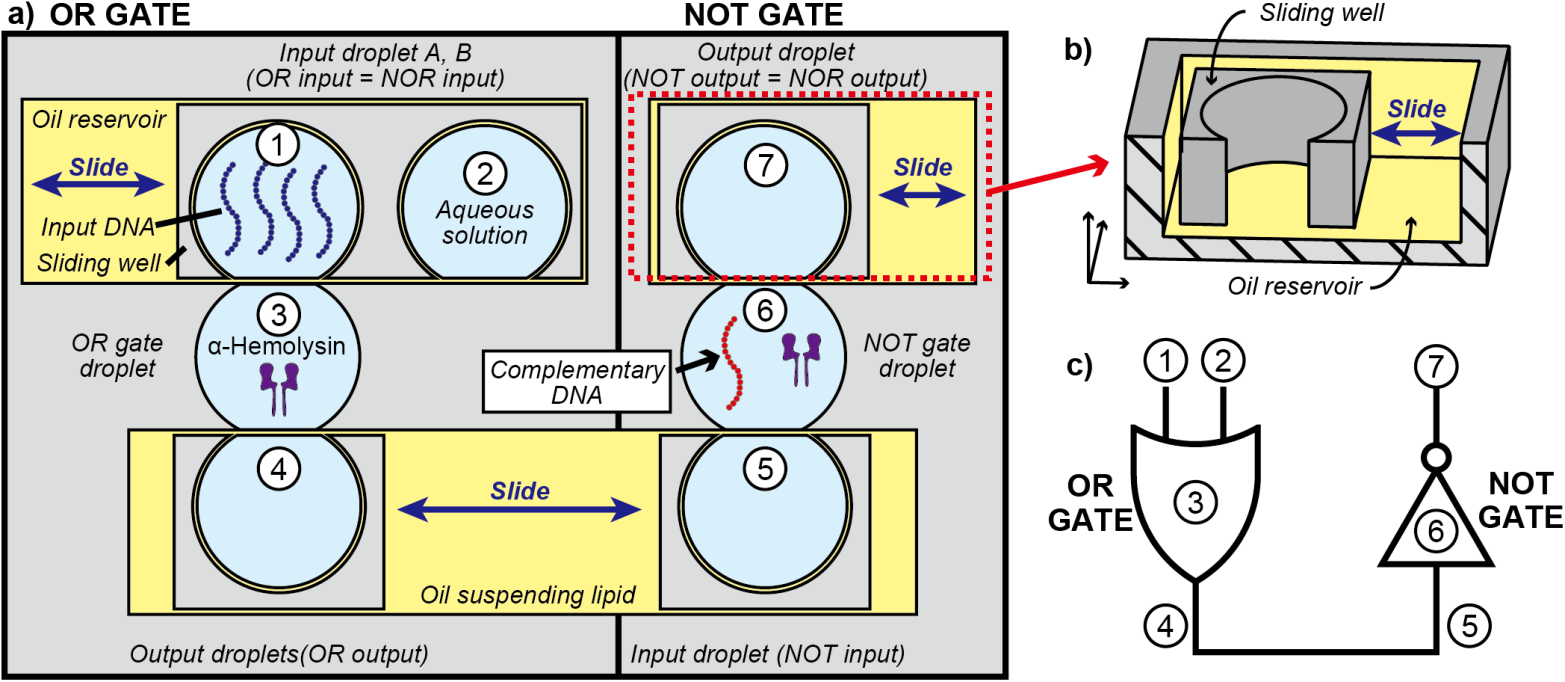
Working principle of the NOR operation. (a) OR gate (left panel): after the OR input droplet A is contacted by the OR gate droplet, the two droplets are fused to initiate mixing of the contents. The same procedure is performed for the OR input droplet B. The OR gate droplet and OR output droplet are fused and mixed. Then, the OR output droplet is slid to the NOT gate region. Note that the OR output droplet is used as the NOT input droplet. NOT gate (right panel): the NOT input droplet is contacted to and fused with the NOT gate droplet. The NOT output droplet is then contacted to the NOT gate droplet. Finally, the binary number in the NOT output droplet is detected by the observation of ssDNA translocation or dsDNA blocking. (b) A perspective view of the sliding well and the oil reservoir. (c) Corresponding logic circuit represented in MIL logic symbols.

The NOR operation was selected as an example of a multi-step DNA logic operation. As shown in Table 1, this operation produces inverted output of the OR gate. Thus, the NOR gate can be constructed by sequentially integrating the OR and NOT gates as depicted in Fig 3. (The detailed working principle of the NOR gate is summarized in the S1 Text).

**Table 1:** Truth table of the NOR gate.

Input A	Input B	Output
0	0	1
0	1	0
1	0	0
1	1	0

The OR operation produces a true output unless both input A and B are false. As shown in Fig 3A (left panel), the OR gate is composed of four droplets: input droplets A and B, OR gate droplet, and output droplet. The binary of the input droplets is defined by whether or not the droplet contains input DNA. Initially, input DNA from the OR input droplets A and B is transferred to the OR gate droplet via the DNA relay mechanism, depending on the input pattern. Then, the input DNA is transferred from the OR gate droplet to the OR output droplet. Hence, the output of the OR operation is stored in the OR output droplet.

As shown in Fig 3A (right panel), the NOT gate is composed of three droplets: input (i.e., OR output), NOT gate, and output droplet. Input DNA in the NOT input droplet, which is slid from the OR operation part, is transferred to the NOT gate droplet via the DNA relay mechanism. In the NOT gate droplet, αHL and a complementary DNA designed to hybridize with input DNA are suspended. By mixing the solutions, input DNA encounters complementary DNA in the NOT operation droplet, and they hybridize into dsDNA. Since the binary number is based on the presence or absence of ssDNA in the output droplet, the hybridization in the logic gate droplet leads to negation of the true binary that was relayed from the input droplet.

## Materials and methods

### Materials

The following chemicals were used without further purification: egg yolk phosphatidylcholine (EggPC; Avanti Polar Lipid, Alabaster, AL, USA); n-decane (Sigma-Aldrich, St. Louis, MO, USA); ethylenediaminetetraacetic acid (EDTA), KCl, K_2_HPO_4_, and KH_2_PO_4_ (Wako Pure Chemical Industries, Ltd., Osaka, Japan); wild-type αHL from Staphylococcus aureus (Sigma-Aldrich); and HPLC-grade DNA oligonucleotide (BEX Co., Ltd., Tokyo, Japan). Ultrapure water with >18 MΩ cm was prepared using a MilliQ system (Merck Millipore, Billerica, MA, USA). Poly(methyl methacrylate) (PMMA) plates with thickness of 0.075, 3.0, and 4.0 mm were obtained from Mitsubishi Rayon (Tokyo, Japan).

### DNA design

For the NOR operation, single-stranded input DNA and complementary DNA molecules were prepared. These ssDNAs were expected to hybridize into dsDNA without any overhang. To avoid the formation of internal secondary structures in a single DNA strand, the input DNA strand was designed using thymine (T) and cytosine (C), whereas the complementary DNA strand was composed of adenine (A) and guanine (G). The base sequences are shown in the S1 Table. The thermodynamic simulation software Nupack (California Institute of Technology, CA, USA)[26] was used to design the DNA; the input DNA and complementary DNA nucleotide sequences were arranged to hybridize into only one type of dsDNA. The input DNA and complementary DNA hybridization was further confirmed by gel electrophoresis. Fluorophore-modified DNA was prepared to evaluate DNA diffusion.

### Electrophoresis

The intended DNA hybridization was confirmed by gel electrophoresis. First, input DNA and complementary DNA were mixed with 10× loading buffer (Takara Bio Inc., Shiga, Japan). Then, 15% acrylamide gel (ATTO Corp., Tokyo, Japan) was prepared using Tris-borate-EDTA buffer (0.089 M Tris boric acid and 2.6 mM EDTA, pH 8.3–8.5; Takara Bio Inc.). The DNA sample was electrophoresed at 150 V for 90 min along with a reference marker [20-bp DNA Ladder (Dye Plus); Takara Bio Inc.]. The gel was visualized by staining in SYBR gold stain (Thermo Fisher Scientific Inc., Waltham, MA, USA) for 15 min. The resultant gel revealed a main band of dsDNA at the same location as the 40-bp reference dsDNA (S1 Fig), inferring that DNA hybridization between input DNA and complementary DNA had occurred as designed.

### Device fabrication

**Fig 4:**
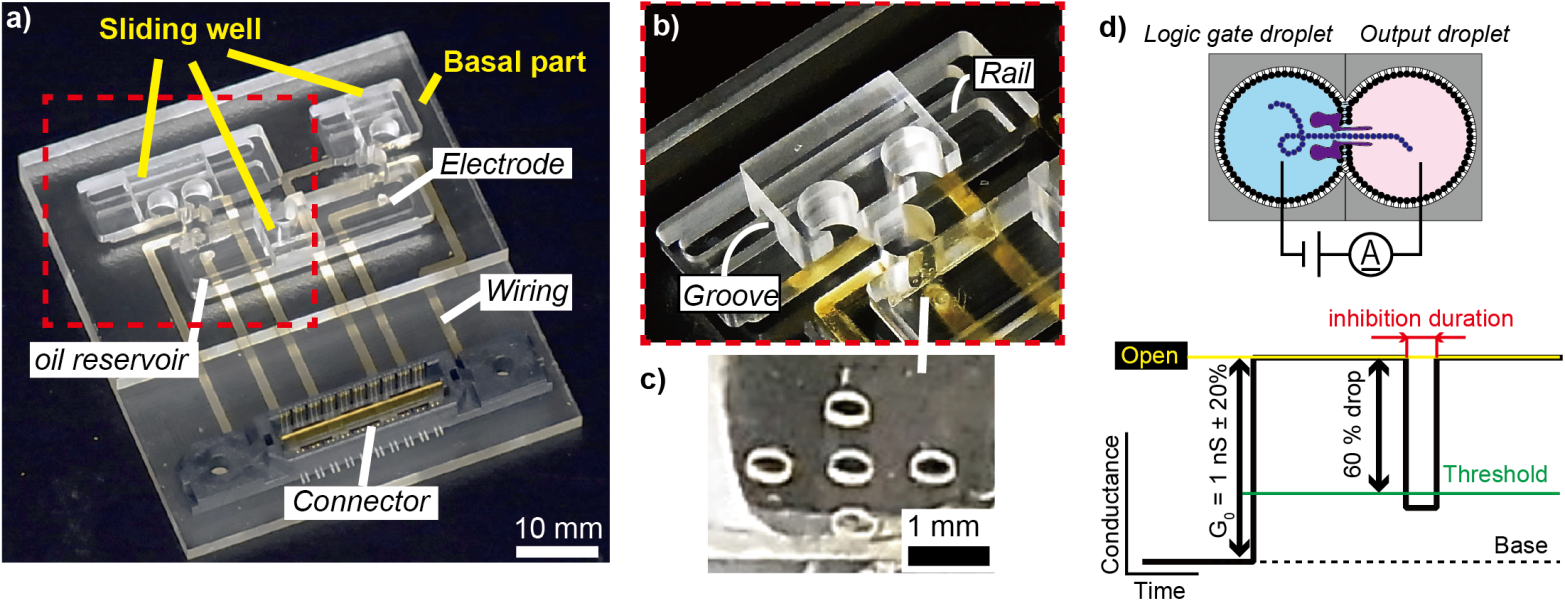
Device images and electrical measurement. (a) Photograph of the device. Ag/AgCl electrodes are places on the bottom of the oil reservoirs and fixed wells, which are connected to an amplifier through Ag wires beneath the reservoir layer. (b) Enlarged view of a sliding well. (c) Poly(methyl methacrylate) (PMMA) separator with a micropore installed between the sliding wells and fixed wells. (d) Electrical measurement between two droplets. The black line indicates the current–time trace. The open channel of α-hemolysin was oriented when the current was increased stepwise with conductance (G_0_) of 1 nS ± 20%. DNA translocation or blocking occurred when the conductance dropped to 60% below the open channel level.

The device for the NOR gate system (Fig 4A) was composed of a basal component and three sliding wells. The basal component included two fixed wells for the logic gate droplets and three oil reservoirs in which the sliding wells were placed. The two fixed wells were placed between the oil reservoirs. A separator film attached to the outlet side of the logic gate well worked to restrict the bilayer area and stabilize the formed bilayer for the electrical readout process (Fig 4C)[19]. A groove and a rail were designed for sliding the wells. Ag/AgCl electrodes were installed to perform electrical fusion and measurements (Fig 4A). Cr/Ag wires were deposited on the backside of the basal component to connect the electrodes with an amplifier19. All parts were fabricated by micromachining PMMA plates (MM-100; Modia Systems, Saitama, Japan). The diameter of all wells and the depth of the reservoirs were set at 4.0 mm and 3.0 mm, respectively.

### Solution of droplets and electrical fusion

A Split-and-Contact device was used to form the bilayer[19]. First, the oil reservoirs were filled with 200 μL of 20 mg/mL EggPC dissolved in n-decane by pipetting. Then, the sliding wells were set in the reservoirs. Next, 20 μL of buffer solution (1.0 M KCl, 2 mM K_2_HPO_4_, 8 mM KH2PO4, and 10 mM EDTA, pH 7.4) containing 3 μM αHL was injected into the OR gate wells by pipetting. 20 μL of buffer solution containing 3 μM αHL and 10 μM complementary DNA was injected into the NOT gate well. For a “true” input condition of the OR gate (or the NOR gate), 40 μM input DNA was infused into the OR input droplet(s) (1 and 2 of Fig 3A); otherwise, the buffer solution was infused. For the “true” input condition of the NOT gate, 10 μM input DNA was infused into the NOT input droplet (5 of Fig 3A) to verify the lone NOT operation.

Electrical fusion was used for mixing the solutions in the two droplets. The lipid bilayer was ruptured with the application of a pulsed voltage at 0.5 V for 1 s using the ZAP function of a patch-clamp amplifier (JET-Bilayer; Tecella LLC, CA, USA). The lipid bilayer rupture was confirmed by current overload.

### Data recording and analysis

As described in the Working Principle section above, the output readout was based on the ionic current signatures via the αHL nanopore incorporated within the lipid bilayer between the logic gate and output droplets; as shown in Fig 2G, ssDNA briefly inhibited the current by translocation leading to a true output, whereas dsDNA caused long current blocking (i.e., false output). The current was acquired using the JET-Bilayer at a sampling rate of 10 kHz and gain of 2.5 mV/pA. The recorded data were digitally filtered at 5 kHz. The clamp voltage was 100 mV. All recordings were performed at 23 ± 1°C in a Faraday cage. The electrical signals obtained were evaluated using Clampfit 10 (Molecular Devices, Sunnyvale, CA, USA). Fig 4D shows a schematic of a current trace over time. The αHL nanopore reconstitution was confirmed by monitoring the stepwise current increase with 1-nS ± 20% conductance. The inhibition events were picked out when the nanopore current was inhibited by more than 60%. The events were classified as either ssDNA translocation or dsDNA blocking with respect to the inhibition duration by using the experimental results (see Results).

## Results

### Determination of mixing duration at the DNA relay

**Fig 5:**
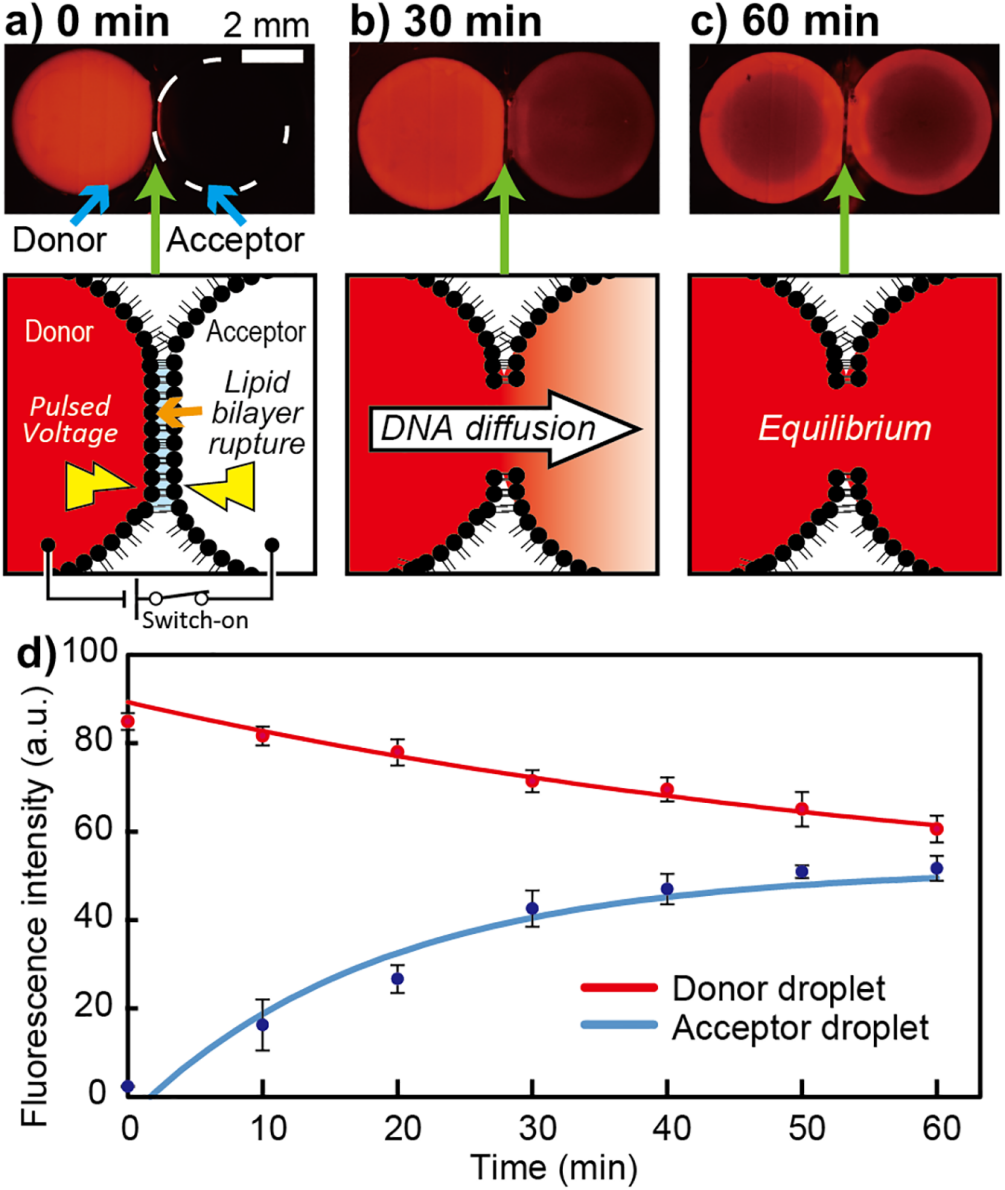
Experiment to determine mixing duration. (a–c) Fluorescence images and corresponding schematics at 0, 30, and 60 min after electrical fusion of the droplets. (d) Time-course monitoring of the fluorescence intensity of the donor and acceptor droplets. Error bars: standard deviations (N = 3).

To determine the mixing duration sufficient for the DNA relay between the droplets, we observed the diffusion of ssDNA between fused droplets (Fig 5A–5C). The two droplets, with and without 40 μM of fluorophore-modified DNA, were prepared in the Split-and-Contact device and named as donor and acceptor droplet, respectively. As shown in Fig 5D, the difference in the fluorescence intensities inside the donor and acceptor droplets decreased over time and the intensities were almost equal at around 1 h. Based on this result, the mixing duration was set to 1 h in the following logic operations.

### Determination of the readout protocols at the logic gates

To properly read the output of logic operation from electric signals, we determined the readout protocols based on discrimination of the ionic current signatures between ssDNA translocation and dsDNA blocking at the nanopore.

We measured the translocation duration of a 40-mer ssDNA through the nanopore. Based on the analysis of 180 translocation events, the average translocation duration (m) and its standard deviation (σ) were 107 ms and 109 ms in our high-gain recording condition, respectively. Assuming that the translocation duration follows a Gaussian distribution, its upper threshold of ssDNA translocation can be set to 435 ms using the equation m + 3σ[24]. Specifically, when the inhibition duration is shorter than the threshold, the event is classified as ssDNA translocation, whereas, when the duration is longer than the threshold, the event is classified as dsDNA blocking. We also obtained the frequency of the translocation events[27–29]. The number of translocation events was collected and the average and standard deviation of the event frequency were derived. The frequency of the event per unit concentration was 2.7 ± 0.7 min^−1^ μM^−1^. The minimum concentration of ssDNA used for logic operation is approximately 10 μM, so that its frequency is expected as 27 ± 7 min^−1^. We therefore set the lower threshold of the frequency of translocation as 6 min^−1^ using the equation m − 3σ.

Based on the experimental results above, we determined readout protocols. For OR gate, the protocol was set as follows (S2 Text). First, the inhibition events are collected for 1 min. Note that each event should fulfill the 60% current drop and the duration below 435 ms. If the number of inhibition events is greater than 6, the output is read out as 1. Otherwise, the output is read out as 0.

For NOT and NOR gates, the readout protocol was set as follows (S3 Text). First, five inhibition events are collected in a single operation and classified into two groups, and each of the following numbers is counted: ssDNA translocation (N_T_) and dsDNA blocking (N_B_). The readout criterion number R_TB_, which is defined as [N_T_ − N_B_]/[N_T_ + N_B_], is then calculated. If R_TB_ is larger than zero, the operation result denotes output 1, whereas, if R_TB_ is less than zero, the result indicates output 0.

### Operations of the single logic gates

The OR and NOT gates were separately demonstrated to confirm that the DNA relay works in single logic gates. The OR operation was performed using the left part of the device depicted in Fig 3A (N = 3 for each input). In the cases of inputs (0, 1), (1, 0), and (1, 1), the numbers of peak-like current inhibition events were respectively 31.4 ± 5.5, 67.0 ± 23.7 and 49.2 ± 10.7 min^−1^ on average, and output 1 was successfully obtained for these three inputs. These readout results validate that the input DNA is successfully relayed from the input droplet to the OR gate droplet through the DNA relay procedure above. In the case of input (0, 0), 1.7 ± 0.6 inhibition signals were detected on average, and output was read out as 0 according to the protocol (S2 Fig). The reason for obtaining the no-zero value of the inhibition events, despite the absence of ssDNA, is considered to be noise of the measurement system. It should be noted that there were no miscalculation in all the three trials of OR operations performed for each input.

The NOT operation was conducted using the right side of the device shown in Fig 3A. In the case of input 0, R_TB_ was 0.73 ± 0.08 (N = 3, S3 Fig) and output was read out as 1 according to the protocol. In the case of input 1, R_TB_ was −0.47 ± 0.08 and output was read out as 0 according to the protocol. The NOT operations succeeded in leading to correct outputs in all the three trials performed for respective inputs. These readout results, in turn, support the premises of our protocol. For the input 0, the complementary DNA in the NOT gate droplet is translocated through the αHL nanopore and peak-like current inhibitions predominantly occur. For the input 1, the input DNA is transferred from the input droplet to the NOT gate droplet and the input DNA and the complementary DNA are spontaneously hybridized into dsDNA, as we designed. As a result, the dsDNA clogs at nanopore and predominantly induces longer inhibitions than 435 ms.

### Operation of the connected logic gate

**Fig 6:**
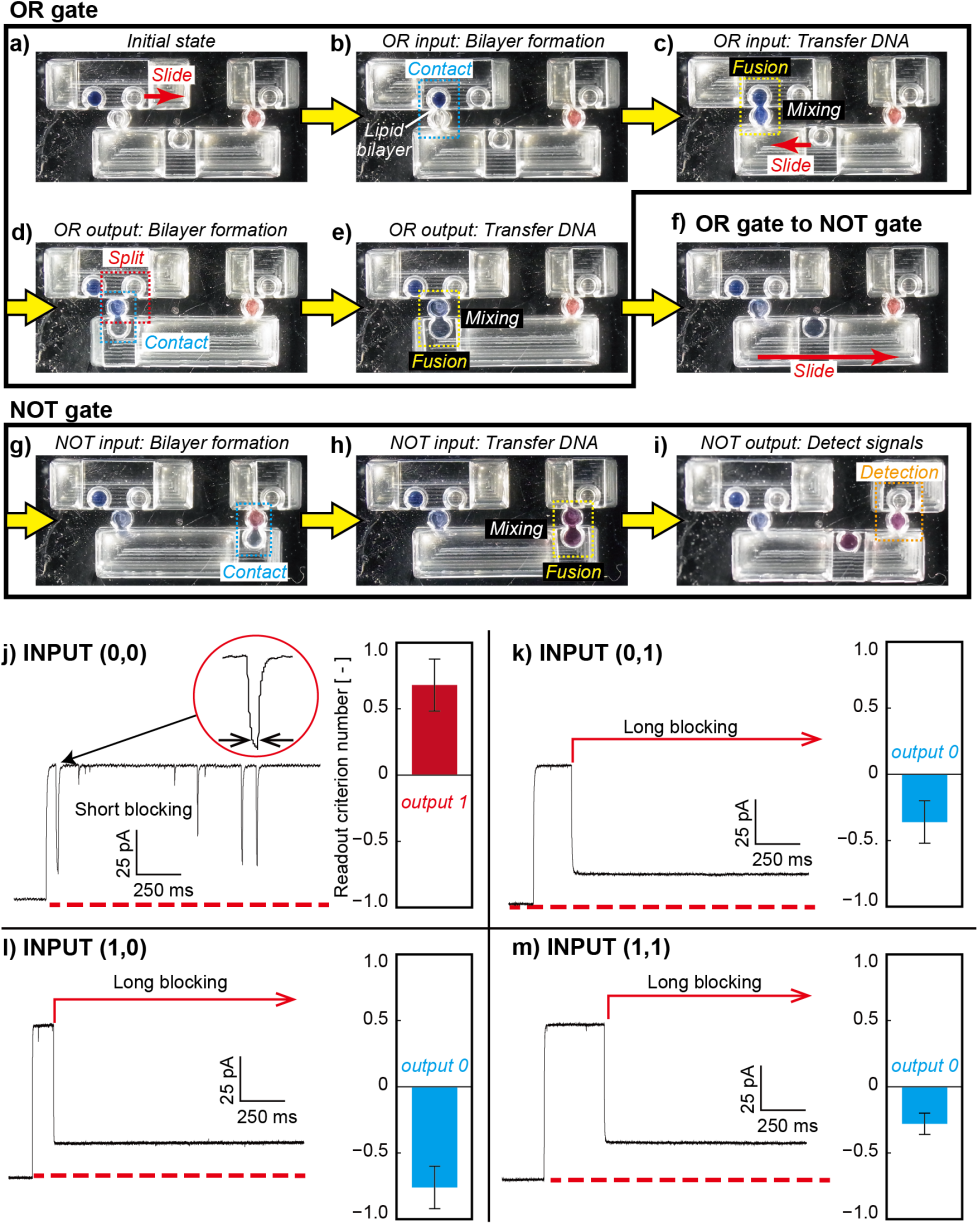
Procedures and results of the NOR operation. (a–i) Procedures of the NOR gate. (j–m) Electrical signals and outputs of the NOR operation. (j) Input (0, 0). Peak-like current inhibitions were predominant, and the readout criterion number (R_TB_) was 0.68 ± 0.20 (i.e., output 1). (k–m) Inputs (0, 1), (1, 0), and (1, 1), respectively. Long current inhibitions were predominant, and the R_TB_ values were −0.36 ± 0.16, −0.76 ± 0.16, and −0.28 ± 0.08, respectively. Thus, these results exhibited output 0.

To confirm whether the proposed DNA relay is able to operate a serial connection of two logic gates, we demonstrated NOR operation. This gate consists of the OR and NOT gates as described above. The connection of the two gates was implemented by a sliding well as shown in Fig 6E–6G.

The representative signals and operation results for each input are summarized in Fig 6J–6M (N = 5 for each input). In the case of input (0, 0), R_TB_ was 0.68 ± 0.20 and it represented output 1 (Fig 6J). There was no miscalculation among five tests for this input. Since no DNA is relayed in this condition, the complementary DNA is the sole DNA in the NOT gate droplet, hence peak-like current inhibitions predominantly occurs. In the case of input (0, 1), (1, 0) and (1, 1), the R_TB_ was −0.36 ± 0.16, −0.76 ± 0.16, and −0.28 ± 0.08, respectively (Fig 6K–6M). These results validate that the input DNA strands are successfully transferred from the OR gate droplet—over several solution mixing processes—into the NOT logic gate droplet, in which produced dsDNA leads to long current inhibitions. The truth result at the OR operation is negated by the hybridization of the input DNA with the complementary DNA in the NOT gate droplet. Almost all the trials, N = 5 for each input, derived the correct output of 0, except the one trial for the input (0, 1). At this miscalculated trial, three ssDNA translocations and two dsDNA blocking were observed (R_TB_ was +0.2). Based on the results, the protocol may be readjusted to improve the operation accuracy. Nevertheless, these results demonstrated that the DNA relay mechanism allowed connection of the sequential OR and NOT operations as expected, and fairly worked out the NOR gate under the readout protocol.

## Discussion

This study confirmed that serial logic gate connections could be implemented by using a series of the DNA relays between the compartments; the DNA relay works by means of electrical fusion and mechanical splitting of droplets. The entire NOR operation was completed within approximately 90 min for each input pattern. Although this duration is reasonable, it is still one order of magnitude longer than the fastest DNA-based logic operation reported[30]. Since the readout duration at the output was approximately 10 min, the operation time was mostly delayed by the molecular diffusion-dependent solution mixing process. Therefore, optimization of the mixing process should considerably reduce the operation time. For example, the mixing could be improved by miniaturization of the droplet volume[31] and/or integration of mixing components such as DNA electrophoresis[32] and droplet mixers[33,34].

In the system presented here, the number of serial connections was limited because the DNA concentration was halved by the relay from one droplet to the other. This dilution causes unbalanced concentrations of input DNA and complementary DNA, leading to the miscalculation of the logic operations. Particularly, this dilution was problematic at the NOR gate where the input DNA experienced three or four mixing processes before the final readout. To improve this limitation for serial connection, DNA amplification by polymerase reaction[16,35], collection of unnecessary DNA by complementary DNA immobilization[36] and/or modification of base sequence in DNA design, e.g. integrating tandem repeat sequence in input DNA, could be considered.

## Conclusion

In summary, by applying electrical fusion and mechanical splitting of droplets, we have developed a microdroplet device with an integrated DNA relay mechanism to connect discrete logic gates. First, we experimentally confirmed that the DNA molecules were adequately relayed by droplet fusion for approximately 1 h. Second, single OR and NOT logic operations were conducted via the internal relay of DNA molecules. Finally, the NOR gate was built as an integration of the OR and NOT gates. The operation results of this study indicated that the DNA relay mechanism enabled a multi-step serial connection of DNA logic gates, which would extend the previous discrete logic gate systems (AND[31,37] and NAND[16,23]) based on droplets. The proposed method is not limited to use with a biological nanopore-based readout but is also applicable to conventional DNA logic operations using enzymatic or hybridization reactions. Droplet-based DNA logic gates created by fusion and splitting potentially allow the integration of multiple DNA logic gates in series and/or parallel, while suppressing unintended crosstalk reactions. As silicon-based logic circuits have obtained sophisticated functionalities through the process of integration, we believe that such expansion in DNA logic circuits will aid in the diagnosis and treatment of cellular diseases.

## Supporting information

S1 Text NOR operation. (DOCX)

S2 Text OR operation protocol. (DOCX)

S3 Text NOT and NOR operation protocol. (DOCX)

S1 Table Nucleotide sequence of the DNA fragments used in this study. (DOCX)

S2 Table Nucleotide sequence of the fluorophore (ROX)-modified DNA. (DOCX)

S1 Fig Representative gel electrophoresis of the hybridized input DNA & complementary DNA sample. (TIF)

S2 Fig Current inhibition and outputs of the OR operation. (a) Input (0, 0). The number of current inhibition events is less than five, i.e., output 0. (b-d) Inputs (0, 1), (1, 0) and (1, 1), respectively. Numerous current inhibition events (e.g., more than five) were recorded, which corresponds to a large number of ssDNA translocating to an output droplet. These operations exhibited output 1. N = 3.
(TIF)

S3 Fig Current inhibition and outputs of the NOT operation. (a) Input 0. Peak-like current inhibition events were dominant, i.e., output 1. (b) Input 1. Long current inhibition events were dominant, i.e., output 0. N = 3.
(TIF)
