# Supplementary material for: Serial DNA relay in DNA logic gates by electrical fusion and mechanical splitting of droplets
Source: PLoS One. 2017 Jul 10;12(7):e0180876. doi: 10.1371/journal.pone.0180876 (PMC5507272; doi:10.1371/journal.pone.0180876)
Supplement: S1 Text — (DOCX) [file pone.0180876.s001.docx]

**S1 Text. NOR operation**

The integration of the OR and NOT gates results in a functional NOR gate as described below. Here, input/output true and false are denoted as input/output 1 and 0, respectively. For example, input (1, 0) stands for input A and B being true and false, respectively.

Input (0, 0): No input DNA is transferred from the OR input droplet to the NOT gate droplet. Therefore, the complementary DNA previously suspended in the NOT gate droplet is transferred to the output droplet, i.e., the output is 1.

Input (0, 1): The input DNA contained in the OR input droplet B is transferred to the NOT gate droplet over three mixing processes. In the NOT gate droplet, the input DNA hybridizes with the complementary DNA, forming dsDNA. Hence, the molecule transferred to the NOT output droplet is dsDNA, i.e., the output is 0.

Input (1, 0): Similar to the input (0, 1), the input DNA contained in the OR input droplet A is transferred to the NOT gate droplet over four mixing processes. The resulting dsDNA is transferred to the NOT output droplet, i.e., the output is 0.

Input (1, 1): Similar to the inputs (0, 1) and (1, 0), the input DNA contained in the OR input droplet A and B is transferred to the NOT gate droplet over three and four mixing processes, respectively. The resulting dsDNA is transferred to the NOT output droplet, i.e., the output is 0.
