# Supplementary material for: Serial DNA relay in DNA logic gates by electrical fusion and mechanical splitting of droplets
Source: PLoS One. 2017 Jul 10;12(7):e0180876. doi: 10.1371/journal.pone.0180876 (PMC5507272; doi:10.1371/journal.pone.0180876)
Supplement: S2 Text — (DOCX) [file pone.0180876.s002.docx]

S2 Text. OR operation protocol

1. Start readout: The readout is executed when α-hemolysin (αHL) is reconstituted to form a nanopore. The reconstitution is confirmed when the current increases stepwise and the conductance G0 is 1 nS ± 20%. If the current increase is higher than 1 nS + 20%, the droplets are split and get into contact. Conductance increase lower than 1 nS + 20% is ignored and the measurement is continued.

2. Count events: A current threshold is set at a conductance line 60% lower than G0. Current inhibition is counted as an event only if the current value decreases below the conductance line, at which point the electrical signal is monitored and recorded for 1 minute. The number of counted events is set as “a”.

3. Output assignment

Output 1: a > 6

Output 0: a ≤ 6
