# Supplementary material for: Serial DNA relay in DNA logic gates by electrical fusion and mechanical splitting of droplets
Source: PLoS One. 2017 Jul 10;12(7):e0180876. doi: 10.1371/journal.pone.0180876 (PMC5507272; doi:10.1371/journal.pone.0180876)
Supplement: S3 Text — (DOCX) [file pone.0180876.s003.docx]

**S3 Text. NOT and NOR operation protocol**

1. Start readout: The readout is executed when αHL is reconstituted as a nanopore. The reconstitution is confirmed when the current increases stepwise and the conductance G_0_ is 1 nS ± 20%. If the increase is higher than 1 nS + 20%, the lipid bilayer is reconstituted by splitting and getting the droplets into contact. A conductance increase lower than 1 nS + 20% is ignored and the measurement is continued.

2. Count events: A current threshold is set at a conductance line 60% lower than G_0_. Current inhibition is counted as an event only if the current value decreases below the conductance line. After the detection of the first five events, the lipid bilayer is reconstituted. In case no inhibition events or insufficient event counts (i.e., less than 5) within two αHL nanopore formation processes are identified, the lipid bilayer is reconstituted. Then, Step 1 and 2 are repeated until five inhibition events are detected.

3. Classify events: Within the defined event duration threshold, the inhibition events are classified and counted into two categories, i.e., ssDNA translocation (number of translocations; N_T_) and dsDNA blocking (number of blockings; N_B_).

4. Output assignment: The readout criterion number [R_TB_ = (N_T_ − N_B_) / (N_T_ + N_B_)] is calculated.

Output 1: R_TB_ > 0

Output 0: R_TB_ < 0
