# Supplementary material for: Serial DNA relay in DNA logic gates by electrical fusion and mechanical splitting of droplets
Source: PLoS One. 2017 Jul 10;12(7):e0180876. doi: 10.1371/journal.pone.0180876 (PMC5507272; doi:10.1371/journal.pone.0180876)
Supplement: S1 Table — (DOCX) [file pone.0180876.s004.docx]

**S1 Table. Nucleotide sequence of the DNA fragments used in this study.**

| DNA | Base sequence |
| --- | --- |
| Input DNA^a^ | 5′-TTTTCCCTTTCCTTTCTTTCCCTTCCTTCTTCCCTCCTCT-3′ (40-mer) |
| Complementary DNA^a^ | 5′-AGAGGAGGGAAGAAGGAAGGGAAAGAAAGGAAAGGGAAAA-3′ (40-mer) |

a: The free energy of hybridization of these DNA fragments, which was calculated using the Nupack software, is −112.0 kJ.
