# Supplementary material for: Serial DNA relay in DNA logic gates by electrical fusion and mechanical splitting of droplets
Source: PLoS One. 2017 Jul 10;12(7):e0180876. doi: 10.1371/journal.pone.0180876 (PMC5507272; doi:10.1371/journal.pone.0180876)
Supplement: S2 Table — (DOCX) [file pone.0180876.s005.docx]

**S2 Table. Nucleotide sequence of the fluorophore (ROX)-modified DNA.**

| DNA | Base sequence |
| --- | --- |
| fluorophore-modified DNA | 5′-TTTTCCCTTTCCTTTCTTTC-3′  (fluorophore ROX attached at the 5' end, 20-mer) |
