# Supplementary figures and images for: Serial DNA relay in DNA logic gates by electrical fusion and mechanical splitting of droplets
Source: PLoS One. 2017 Jul 10;12(7):e0180876. doi: 10.1371/journal.pone.0180876 (PMC5507272; doi:10.1371/journal.pone.0180876)

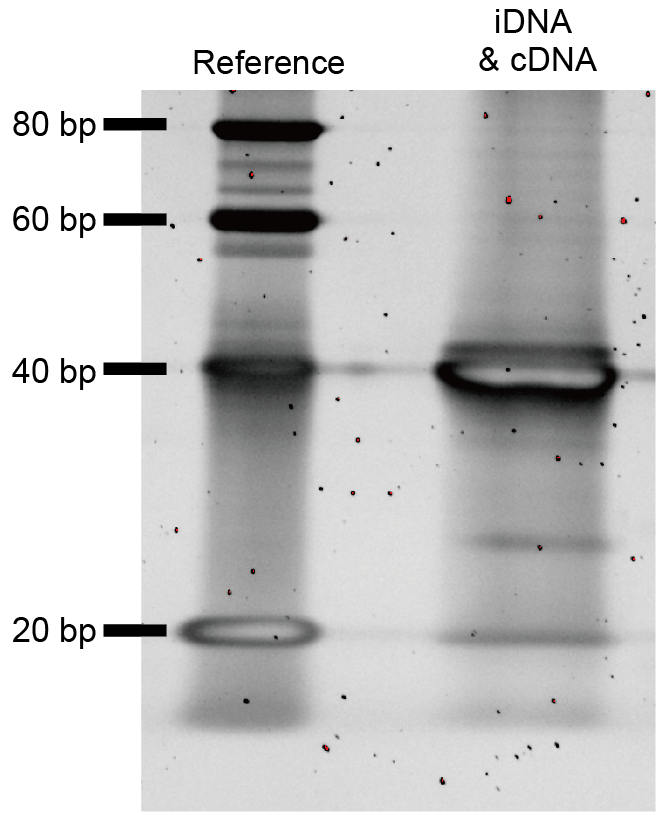

Supplement: S1 Fig — (TIF) [file pone.0180876.s006.tif]

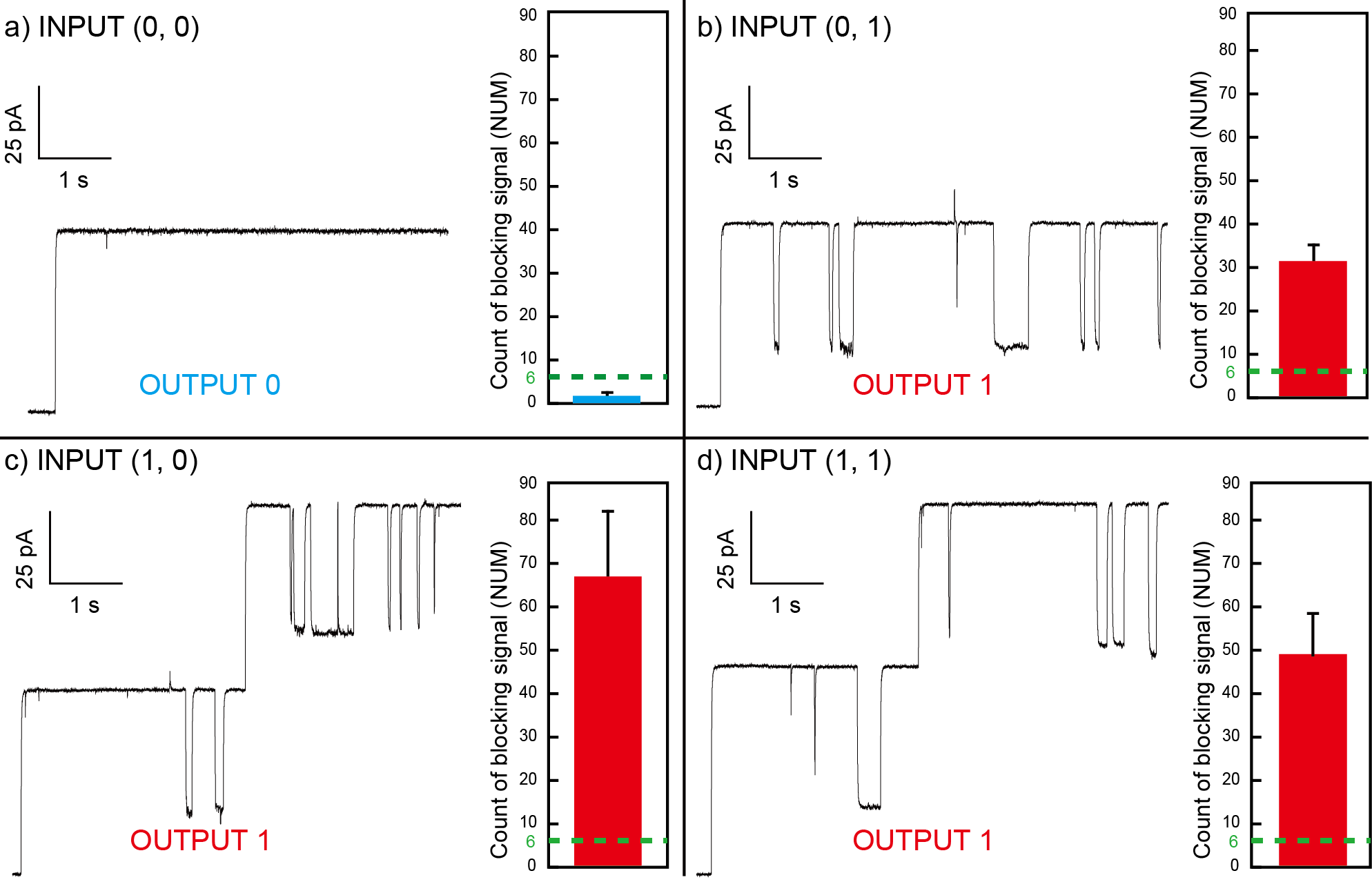

Supplement: S2 Fig — (a) Input (0, 0). The number of current inhibition events is less than five, i.e., output 0. (b-d) Inputs (0, 1), (1, 0) and (1, 1), respectively. Numerous current inhibition events (e.g., more than five) were recorded, which corresponds to a large number of ssDNA translocating to an output droplet. These operations exhibited output 1. N = 3. (TIF) [file pone.0180876.s007.tif]

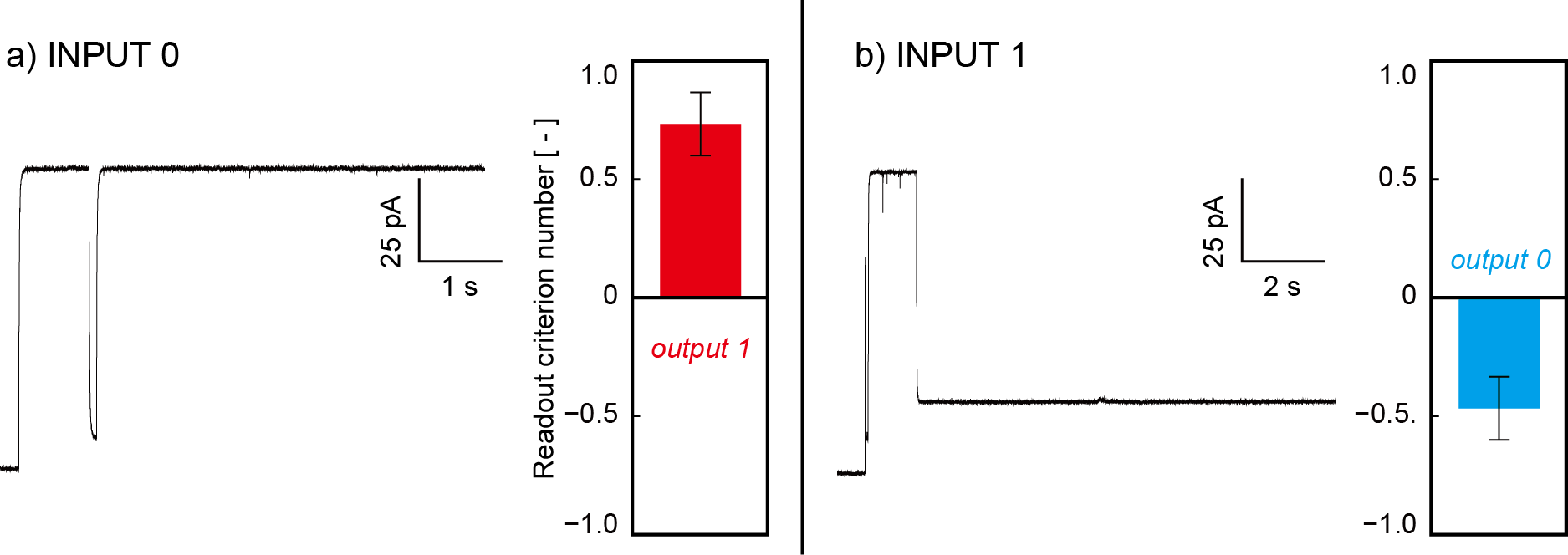

Supplement: S3 Fig — (a) Input 0. Peak-like current inhibition events were dominant, i.e., output 1. (b) Input 1. Long current inhibition events were dominant, i.e., output 0. N = 3. (TIF) [file pone.0180876.s008.tif]
